# Grafted Semiflexible Nunchucks with a Magnetic Bead Attached to the Free End

**DOI:** 10.3390/polym14040695

**Published:** 2022-02-11

**Authors:** Mohammadhosein Razbin, Panayotis Benetatos

**Affiliations:** 1 Department of Energy Engineering and Physics, Amirkabir University of Technology, Tehran 14588, Iran; 2 Department of Physics, Kyungpook National University, 80 Daehakro, Bukgu, Daegu 41566, Korea

**Keywords:** semiflexible polymers, hinged polymers, bimodality, magnetometry, signal enhancement

## Abstract

Semiflexible nunchucks are block copolymers, which consist of two long blocks of high bending stiffness jointed together by a short block of low bending stiffness. Semiflexible nunchucks that consist of two DNA nanorods jointed by a short segment of double-stranded (ds) DNA and confined in two dimensions have been used in recent experiments by Fygenson and coworkers as a tool to magnify the bending fluctuations of the linking dsDNA, which in turn are used to deduce the persistence length of dsDNA. In a recent theoretical analysis, we showed that in a semiflexible nunchuck with one end grafted, the fluctuations of the position of the free end that is transverse to the grafting direction exhibit a pronounced bimodality, provided that the bending stiffness of the hinge is not very large. In this article, we theoretically analyse a grafted semiflexible nunchuck with a magnetic bead attached to its free end. We show that a transverse magnetic field induces an asymmetry in the bimodal distribution of the transverse fluctuations of the free end. This asymmetry is very sensitive to interactions with a magnetic field and, in principle, could be used in magnetometry (the measurement of a magnetic field or the magnetic moment of the bead). We also investigate how the response of the bimodal distribution of the transverse fluctuations of the free end to a magnetic field depends on the bending stiffness of the nunchuck hinge. In addition, we analyse the closely related systems of a single filament and two filaments jointed at a kink point with one end grafted and the other end attached to a magnetic bead.

## 1. Introduction

Semiflexible nunchucks are block copolymers consisting of two long blocks of high bending stiffness linked together by a short block of lower bending stiffness. Such nanostructures have been manufactured using DNA nanotubes linked by a segment of dsDNA [1,2]. The DNA nanotubes are fluorescently labeled and confined in two dimensions between two glass plates. Their fluctuations are directly visualised and act as a magnification of the bending fluctuations of the short dsDNA segment. The spread of the distribution of the bending angle is used to deduce the persistence length of the dsDNA.

The conformational fluctuations of a semiflexible nunchuck in two dimensions are amenable to analytical treatment [3]. Assuming one end to be grafted, we can calculate (up to a numerical integration) the probability distribution of the position of the free end. We assume that the contour length of the linking block is negligible compared to the contour length of the two arms, and we treat it as a harmonic orientational spring that is characterized by a bending stiffness. The probability distribution of the transverse position of the free end (after integrating out the longitudinal position) assumes a unimodal or bimodal form, depending on the bending stiffness of the hinge between the two arms. For large values of bending stiffness, the distribution is unimodal with one peak and as the bending stiffness decreases, the distribution flattens and eventually develops a pronounced bimodality with two peaks. In order to understand the origin of this emergent bimodality, let us consider the limiting case of a grafted nunchuck having two perfectly rigid arms jointed by a perfectly soft hinge. In that case, all orientations of the fluctuating arm are equally probable, and the same is true for the position of the free end. After integrating out the longitudinal position, the probability density of the transverse position exhibits a pronounced bimodality. A similar bimodality has been predicted theoretically and observed in simulations of semiflexible homopolymers with a contour length of the same order as its persistence length [4,5,6,7]. This bimodality, or lack thereof, can be used as a rough estimate for the bending stiffness of the linking polymer that acts as a hinge.

Magnetic beads attached to polymers, such as nucleic acids or proteins, are used in order to exert forces or torques and probe their conformations at the single-molecule level (magnetic tweezers) [8,9,10,11,12]. In addition to their use in single-molecule experiments, magnetic beads are used to analyse the elasticity of cells or extracellular matrices [13,14]. In this article, we consider a grafted semiflexible nunchuck with a magnetic bead attached to its free end and two closely related systems. Our theoretical analysis is based on the weakly bending approximation of the worm-like chain (WLC). Firstly, we consider a single WLC at the stiff limit with one end grafted and the other end attached to a magnetic bead. We also consider a kinked grafted system of two weakly bending WLCs rigidly jointed at a kinked joint with one end grafted and the other end attached to a magnetic bead. The main focus of this study is the grafted semiflexible nunchuck. In the absence of magnetic interaction, the probability distribution of the transverse fluctuations of the free end is symmetrical for an aligning hinge. Interaction with a magnetic field that is pointing in a direction different from that of the grafted arm breaks this symmetry. The ensuing asymmetrical distribution is sensitive to the parameters of the system, such as the magnetic interaction energy and the bending stiffness of the hinge. We show that this dependence could, in principle, be used in magnetometry or as an alternative method to determine the bending stiffness of the hinge polymer. Magnetometry involves two types of measurements. If the magnetic field is known, we can determine the magnetic moment of the bead. In the other case when the magnetic moment of the bead is known, we can determine the magnetic field.

The article is organised as follows. In Section 2, we consider a single grafted WLC at the stiff (weakly bending) limit. We review its conformational properties and then we consider the effect of a magnet attached to its free end. In Section 3, we consider two WLCs at the stiff limit that are jointed by a stiff kink. One end of the system is grafted and the free end has a magnetic bead attached to it. In Section 4, we consider the case of a grafted semiflexible nunchuck with a magnetic bead and focus on the effect of the magnetic interaction on the bimodal distribution of the transverse fluctuations of the free end. We conclude and summarize in Section 5. Some complicated formulas are presented in the Appendix A.

## 2. Single WLC with a Magnetic Bead at the Stiffness (Weakly Bending) Limit

### 2.1. The Positional–Orientational Propagator of a WLC at the Stiff Limit

**Figure 1:**
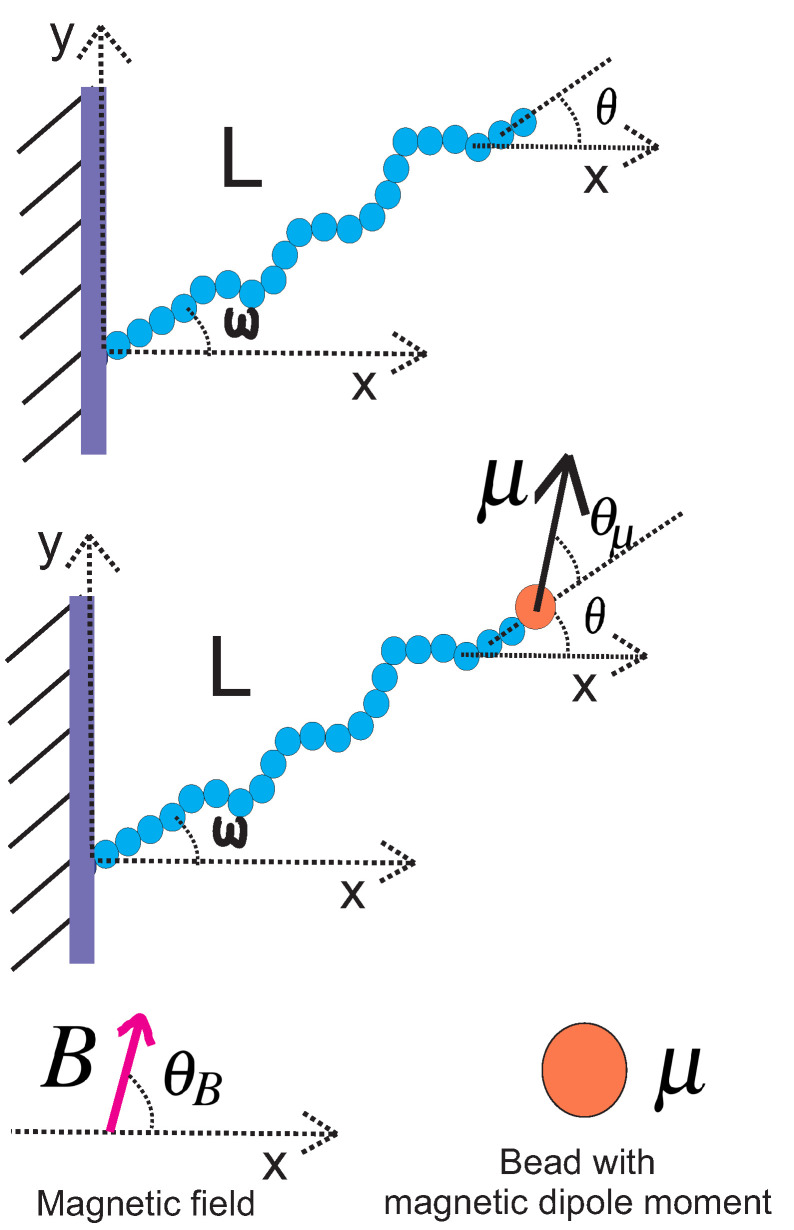
**Upper panel**: A typical configuration of a rather stiff grafted semiflexible filament in the presence of thermal fluctuations. The persistence length of the filament is $l_{p}$ and it has contour length *L*. The filament is grafted in a substrate with grafting angle ω. **Lower panel**: The same filament with one tip attached to a magnetic bead.

In this subsection, we review the behaviour of a grafted WLC in two dimensions, as shown in Figure 1, at the weakly bending stiff limit [3]. Due to the large value of bending rigidity, the persistence length is much greater than the total contour length $L\ll l_{P}$ and the deflection away from the grafting direction is small, so that sin(θ−ω)≈θ−ω and cos(θ−ω)≈1. The conditional probability density to find the end point of the chain at position (x,y) with orientation θ, given that it is grafted at position $\left(x_{0},y_{0}\right)$ with orientation ω, is denoted by $G_{L,l_{p}}\left(x,y,\theta |x_{0},y_{0},\omega \right)$ and is called the positional–orientational propagator because it obeys the Chapman–Kolmogorov equation. In the weakly bending regime, the propagator is calculated in closed analytic form [3,5,15,16,17,18,19]:

(1) $$\begin{array}{c} G_{L,l_{p}}\left(x,y,\theta |x_{0},y_{0},\omega \right)=\frac{1}{N_{G}}\exp[-\frac{3l_{p}}{L^{3}}(\left(y-y_{0}\right)\cos\left(\omega \right)\\ -\left(x-x_{0}\right)\sin\left(\omega \right){)}^{2}-\frac{l_{p}}{L}{\left(\theta -\omega \right)}^{2}]\\ \times \exp[\frac{3l_{p}}{L^{2}}\left(\left(y-y_{0}\right)\cos\left(\omega \right)-\left(x-x_{0}\right)\sin\left(\omega \right)\right)\left(\theta -\omega \right)]\\ \times \delta [\left(x-x_{0}\right)\cos\left(\omega \right)+\left(y-y_{0}\right)\sin\left(\omega \right)-L]\;, \end{array}$$

where δ(x) is the Dirac δ-function and the normalization factor $N_{G}$ is determined by the condition

(2) $$\begin{array}{c} \int \int \int dxdyd\theta \;G_{L,l_{p}}\left(x,y,\theta |x_{0},y_{0},\omega \right)=1\;. \end{array}$$

In the remainder of this article, we use the notation $\int \equiv \int_{-\infty }^{+\infty }$ for the sake of simplicity. Using Equation (1), we can easily calculate the probability density of the *x* component of the position of the free end point.

(3) $$\begin{array}{cc} P_{x}^{\prime }\left(x\right) & =\int \int dyd\theta G_{L,l_{p}}\left(x,y,\theta |0,0,\omega \right)\\ & =\sqrt{\frac{3l_{p}}{4\pi L^{3}\sin^{2}\left(\omega \right)}}\exp\left(-\frac{3l_{p}{\left(x-L\cos\left(\omega \right)\right)}^{2}}{4L^{3}\sin^{2}\left(\omega \right)}\right)\;. \end{array}$$

The probability density of the *y* component of the position of the free end point is:

(4) $$\begin{array}{cc} P_{y}^{\prime }\left(y\right) & =\int \int dxd\theta G_{L,l_{p}}\left(x,y,\theta |0,0,\omega \right)\\ & =\sqrt{\frac{3l_{p}}{4\pi L^{3}\cos^{2}\left(\omega \right)}}\exp\left(-\frac{3l_{p}{\left(y-L\sin\left(\omega \right)\right)}^{2}}{4L^{3}\cos^{2}\left(\omega \right)}\right)\;. \end{array}$$

In addition, the probability density of the tangent vector orientation at the free end point turns out to be:

(5) $$\begin{array}{cc} P_{\omega }^{\prime }\left(\theta \right) & =\int \int dxdyG_{L,l_{p}}\left(x,y,\theta |0,0,\omega \right)\\ & =\sqrt{\frac{l_{p}}{4\pi L}}\exp\left(-\frac{l_{p}{\left(\theta -\omega \right)}^{2}}{4L}\right)\;. \end{array}$$

We point out that even though Equations (3) and (4) rely on the validity of the weakly bending approximation, Equation (5) is exact and valid for any value of bending stiffness. Next, we attach a bead with a magnetic dipole moment to the tip of the filament (see Figure 1).

### 2.2. Grafted Stiff WLC with One End Attached to a Magnetic Bead

We consider a grafted weakly bending WLC with a bead having a magnetic dipole moment $\vec{\mu }$ attached to the free end and exposed to a uniform magnetic field $\vec{B}$. The magnetic bead in this article was assumed to be a point particle with a magnetic dipole moment. Both vectors are assumed to have only *x* and *y* components. The magnetic interaction energy is $-\vec{\mu }\cdot \vec{B}=-\mu B\cos\left(\theta +\theta_{\mu }-\theta_{B}\right)$. The orientation of the magnetic dipole deviates by a fixed value $\theta_{\mu }$ from the orientation of the tip of the filament θ. Therefore, the orientation of the magnetic bead was $\theta +\theta_{\mu }$. The orientation of the magnetic field $\theta_{B}$ is fixed (see Figure 1). This interaction affects the filament conformations through the Boltzmann weight $\exp[\vec{\mu }\cdot \vec{B}/\left(k_{B}T\right)]$.

The probability density of the *x* component of the end point position at the Gaussian limit is given by the following expression:

(6) $$\begin{array}{ccc} P_{x}\left(x\right) & = & \int \int dyd\theta G_{L,l_{p}}\left(x,y,\theta |0,0,\omega \right)\\ & & \times \frac{1}{N_{B}}\exp\left(K_{B}\cos\left(\theta +\theta_{\mu }-\theta_{B}\right)\right)\\ & = & \frac{1}{N_{x}}\exp\left(A_{x}\right)\;, \end{array}$$

where $N_{x}$ is the normalization factor and $A_{x}$ is given by Equation (A2) in the Appendix A. $K_{B}=\frac{\mu B}{k_{B}T}$ is the dimensionless strength of the magnetic energy relative to the thermal energy.

The probability density of the *y* component of the free end point position at the weakly bending (Gaussian) limit is:

(7) $$\begin{array}{ccc} P_{y}\left(y\right) & = & \int \int dxd\theta G_{L,l_{p}}\left(x,y,\theta |0,0,\omega \right)\\ & & \times \frac{1}{N_{B}}\exp\left(K_{B}\cos\left(\theta +\theta_{\mu }-\theta_{B}\right)\right)\\ & = & \frac{1}{N_{y}}\exp\left(A_{y}\right)\;, \end{array}$$

where $N_{y}$ is the normalization factor and $A_{y}$ is given by Equation (A6) in the Appendix A. In addition, the probability density of the orientation of the free end point is given by:

(8) $$\begin{array}{ccc} P_{\omega }\left(\theta \right) & = & \int \int dydxG_{L,l_{p}}\left(x,y,\theta |0,0,\omega \right)\\ & & \times \frac{1}{N_{B}}\exp\left(K_{B}\cos\left(\theta +\theta_{\mu }-\theta_{B}\right)\right)\\ & = & \frac{1}{N_{\theta }}\exp\left(-\frac{l_{p}{\left(\theta -\omega \right)}^{2}}{4L}+K_{B}\cos\left(\theta +\theta_{\mu }-\theta_{B}\right)\right)\;. \end{array}$$

**Figure 2:**
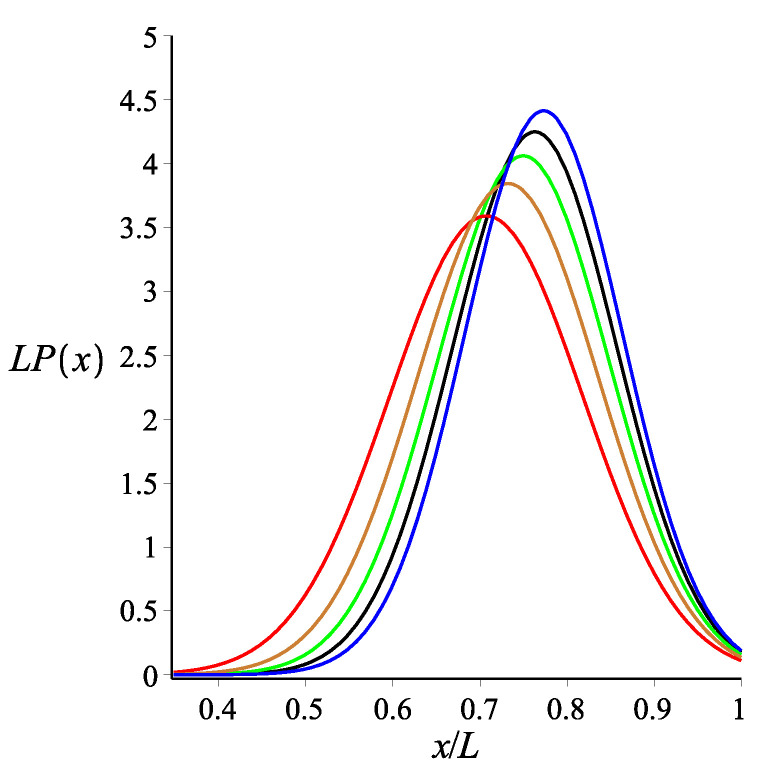
The probability density of the *x* coordinate of the position of the tip of a single grafted filament with a magnetic bead, as shown by Equation (6). The red, gold, green, black and blue colours correspond to $K_{B}=0$, $K_{B}=3$, $K_{B}=6$, $K_{B}=9$ and $K_{B}=12$, respectively. The fixed parameters for all curves are: L=1 μm; $l_{p}=27$ μm; $\omega =\frac{\pi }{4}$; $\theta_{B}=\frac{\pi }{8}$; and $\theta_{\mu }=0$.

**Figure 3:**
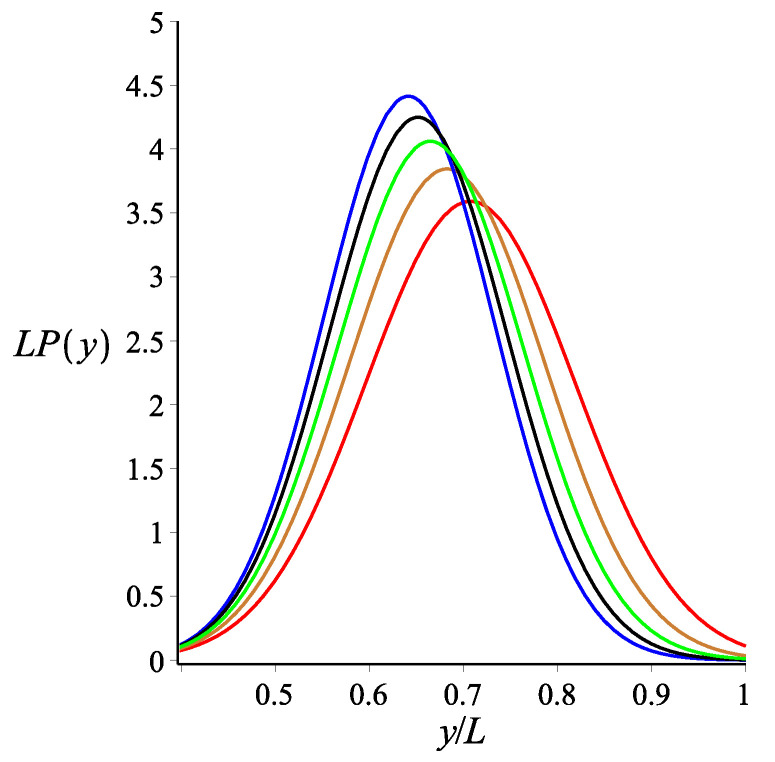
The probability density of the *y* coordinate of the position of the tip of a single grafted filament with a magnetic bead, as shown by Equation (7). The red, gold, green, black and blue colours correspond to $K_{B}=0$, $K_{B}=3$, $K_{B}=6$, $K_{B}=9$ and $K_{B}=12$, respectively. The fixed parameters for all curves are: L=1 μm; $l_{p}=27$ μm; $\omega =\frac{\pi }{4}$; $\theta_{B}=\frac{\pi }{8}$; and $\theta_{\mu }=0$.

**Figure 4:**
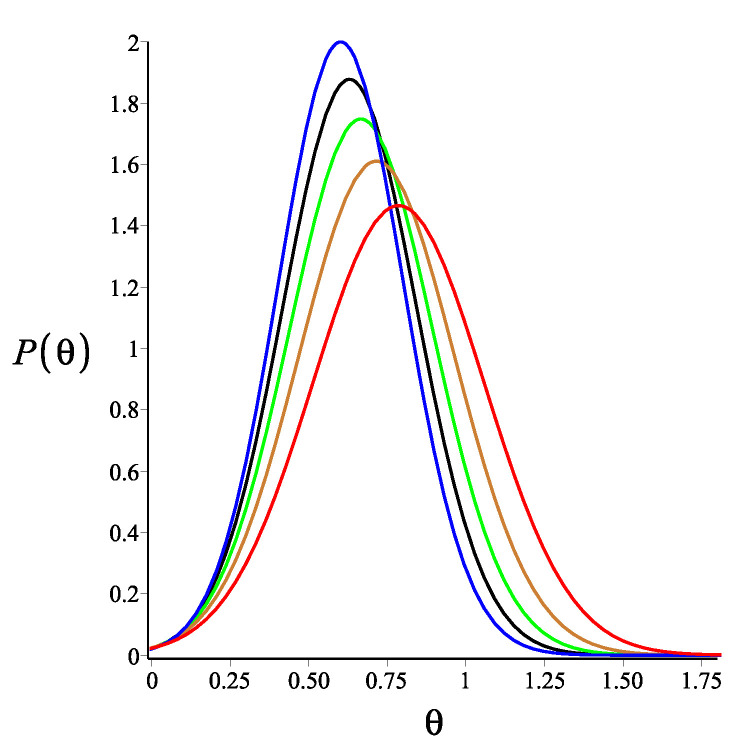
The probability density of the orientation of the tip of a single grafted filament with a magnetic bead, as shown by Equation (8). The red, gold, green, black and blue colours correspond to $K_{B}=0$, $K_{B}=3$, $K_{B}=6$, $K_{B}=9$ and $K_{B}=12$, respectively. The fixed parameters for all curves are: L=1 μm; $l_{p}=27$ μm; $\omega =\frac{\pi }{4}$; $\theta_{B}=\frac{\pi }{8}$; and $\theta_{\mu }=0$.

Here, Equation (8) is calculated using the weakly bending approximation, but it is generally valid due to the fact that Equation (5) is exact. As a consistency check, we look at the limit of $K_{B}=0$. At this limit, Equations (6)–(8) reduce to Equations (3)–(5), respectively. The effect of the magnetic interaction on the filament conformations is shown in Figure 2, Figure 3 and Figure 4.

## 3. Two Weakly Bending WLCs Jointed at a Stiff Kink Point with One End Attached to a Magnetic Bead

**Figure 5:**
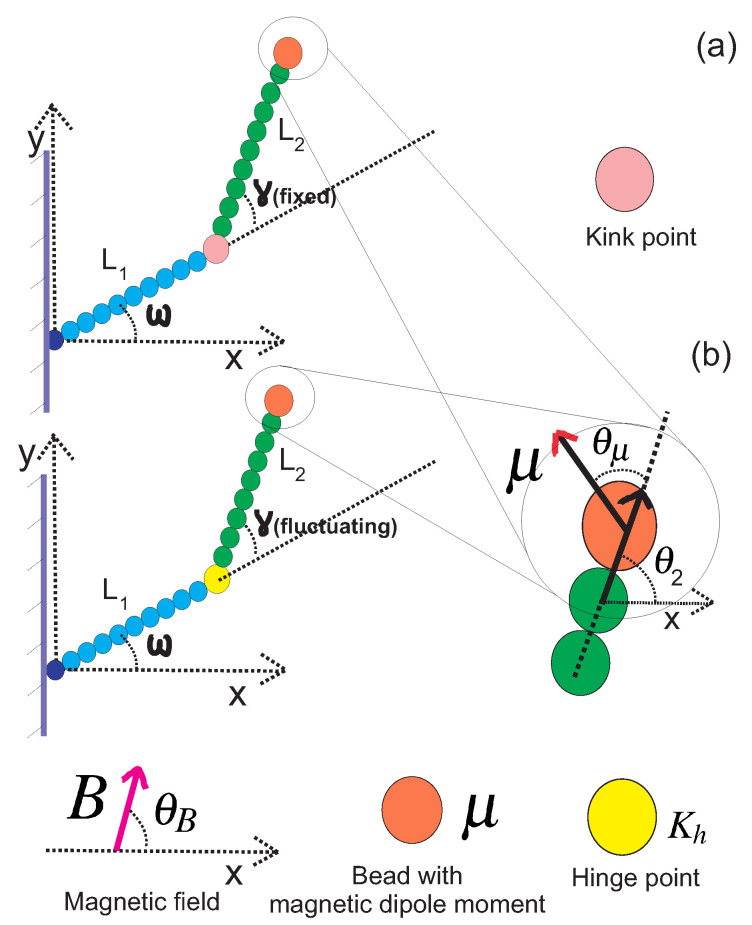
**Upper panel** (**a**): A configuration of two jointed weakly bending semiflexible filaments. The stiff joint (kink point) has a kink angle γ. The first filament has contour length $L_{1}$ and persistence length $l_{p1}$. The second filament has contour length $L_{2}$ and persistence length $l_{p2}$. The first filament is grafted onto a fixed substrate with a grafting angle ω. **Lower panel** (**b**): A configuration of two jointed semiflexible filaments with a hinge point. This differs from the system in the upper panel in that the kink angle γ fluctuates around an average value $\gamma_{0}$. The hinge point has a rotational (bending) stiffness $K_{h}$.

In this Section, we consider two WLCs, both at the stiff limit but they can have different persistence lengths. They are jointed at a kink point and the kink angle γ is fixed (it does not fluctuate). In the upper panel of Figure 5, we show the configuration of such a grafted kinked pair of arms. The first arm is grafted onto the substrate with orientation ω, and it has persistence length $l_{p1}$ and contour length $L_{1}$. The second arm is attached to the end point of the first arm at the kink point and it has persistence length $l_{p2}$ and contour length $L_{2}$. We label the end point of the first arm with the number one, which is also the kink point. We label the end point of the second arm of the structure with the number two. The end point of the structure is the same as the end point of the second arm. By concatenating the propagators associated with the two arms, we calculate the probability density to find the *y* component of the position of the end point of the kinked structure at a given value $y_{2}$:

(9) $$\begin{array}{ccc} P_{ky}\left(y_{2}\right)= & & \int \int \int dx_{1}dy_{1}d\theta_{1}G_{L_{1},l_{p1}}\left(x_{1},y_{1},\theta_{1}|0,0,\omega \right)\\ & & \times \int \int dx_{2}d\theta_{2}G_{L_{2},l_{p2}}\left(x_{2},y_{2},\theta_{2}|x_{1},y_{1},\theta_{1}+\gamma \right)\\ & & \times \frac{1}{N_{B}}\exp\left(K_{B}\cos\left(\theta_{2}+\theta_{\mu }-\theta_{B}\right)\right)\;. \end{array}$$

and

(10) $$\begin{array}{ccc} P_{k\theta }\left(\theta_{2}\right) & = & \int \int \int dx_{1}dy_{1}d\theta_{1}G_{L_{1},l_{p1}}\left(x_{1},y_{1},\theta_{1}|0,0,\omega \right)\\ & & \times \int \int dx_{2}dy_{2}G_{L_{2},l_{p2}}\left(x_{2},y_{2},\theta_{2}|x_{1},y_{1},\theta_{1}+\gamma \right)\\ & & \times \frac{1}{N_{B}}\exp\left(K_{B}\cos\left(\theta_{2}+\theta_{\mu }-\theta_{B}\right)\right)\;. \end{array}$$

By performing the integrals in Equation (9), we obtain an analytic expression for $P_{ky}\left(y_{2}\right)$ at the Gaussian limit:

(11) $$P_{ky}\left(y_{2}\right)=\frac{1}{N_{ky}}\exp\left(A_{ky}\right)\;,$$

where $A_{ky}$ is given in the Appendix A.

By performing the Gaussian integrals in Equation (10), we obtain the probability distribution of the orientational fluctuations of the free end:

(12) $$P_{k\theta }\left(\theta_{2}\right)=\frac{1}{N_{k\theta }}\exp\left(K_{B}\cos\left(\theta_{2}+\theta_{\mu }-\theta_{B}\right)-\frac{l_{p1}l_{p2}{\left(\theta_{2}-\omega -\gamma \right)}^{2}}{4L_{2}l_{p1}+4L_{1}l_{p2}}\right)\;.$$

In the weakly bending approximation, all integrals are Gaussian. As a consistency check, we look at two limiting cases. In the first case, γ=0, $L_{1}=L_{2}=\frac{L}{2}$ and $l_{p}=l_{p1}=l_{p2}$, while in the second case, $L_{1}=0$, γ=0 and $l_{p}=l_{p2}$. In both cases, the two probability density functions of Equations (11) and (12) reduce to Equations (7) and (8), respectively, which correspond to a single filament with length *L*.

## 4. Two Weakly Bending WLCs Jointed at a Hinge Point (Semiflexible Nunchuck) with One End Attached to a Magnetic Bead

In this Section, we consider the most interesting case: that of a grafted semiflexible nunchuck with a magnetic bead at the free end, as shown in the lower panel of Figure 5. We treat the linking middle block as a point hinge with a bending stiffness (orientational spring). The only approximation concerning the middle block is the assumption that it has a negligible length compared to the length of the two arms. The Gaussian distribution of its bending fluctuations is exact, and it holds irrespective of the persistence length of the linking polymer segment. By concatenating the propagators associated with the two arms, we calculate the probability density to find the *y* component of the position of the end point of the hinged structure at a given value $y_{2}$:

(13) $$\begin{array}{ccc} P_{ky}\left(y_{2}\right) & = & \int \int \int \int d\gamma \;dx_{1}dy_{1}d\theta_{1}G_{L_{1},l_{p1}}\left(x_{1},y_{1},\theta_{1}|0,0,\omega \right)\\ & & \times \int \int dx_{2}d\theta_{2}G_{L_{2},l_{p2}}\left(x_{2},y_{2},\theta_{2}|x_{1},y_{1},\theta_{1}+\gamma \right)\\ & & \times \frac{1}{N_{B}}\exp\left(K_{B}\cos\left(\theta_{2}+\theta_{\mu }-\theta_{B}\right)\right)P_{h}\left(\gamma \right)\;, \end{array}$$

where

(14) $$P_{h}\left(\gamma \right)=\sqrt{\frac{K_{h}}{2\pi }}\exp\left(-\frac{K_{h}}{2}{\left(\gamma -\gamma_{0}\right)}^{2}\right)\;,$$

$K_{h}=\frac{k_{h}}{k_{B}T}$ is the ratio of the bending stiffness of the hinge point to the thermal energy and $\gamma -\gamma_{0}$ is the angle deviation from the rest angle of the hinge point. Additionally:

(15) $$\begin{array}{ccc} P_{k\theta }\left(\theta_{2}\right) & = & \int \int \int \int d\gamma dx_{1}dy_{1}d\theta_{1}G_{L_{1},l_{p1}}\left(x_{1},y_{1},\theta_{1}|0,0,\omega \right)\\ & & \times \int \int dx_{2}dy_{2}G_{L_{2},l_{p2}}\left(x_{2},y_{2},\theta_{2}|x_{1},y_{1},\theta_{1}+\gamma \right)\\ & & \times \frac{1}{N_{B}}\exp\left(K_{B}\cos\left(\theta_{2}+\theta_{\mu }-\theta_{B}\right)\right)P_{h}\left(\gamma \right)\;. \end{array}$$

By performing five of the integrals in Equation (13), we obtain a single-integral expression for $P_{hy}\left(y_{2}\right)$ at the Gaussian limit (the weakly bending limit for the two arms):

(16) $$P_{hy}\left(y_{2}\right)=\int P_{ky}\left(y_{2}\right)P_{h}\left(\gamma \right)d\gamma \;.$$

Similarly, we perform five of the Gaussian integrals in Equation (15) and obtain the following single-integral expression for the orientational fluctuations of the free end:

(17) $$P_{h\theta }\left(\theta_{2}\right)=\int P_{k\theta }\left(\theta_{2}\right)P_{h}\left(\gamma \right)d\gamma \;.$$

These integrals have to be evaluated numerically.

**Figure 6:**
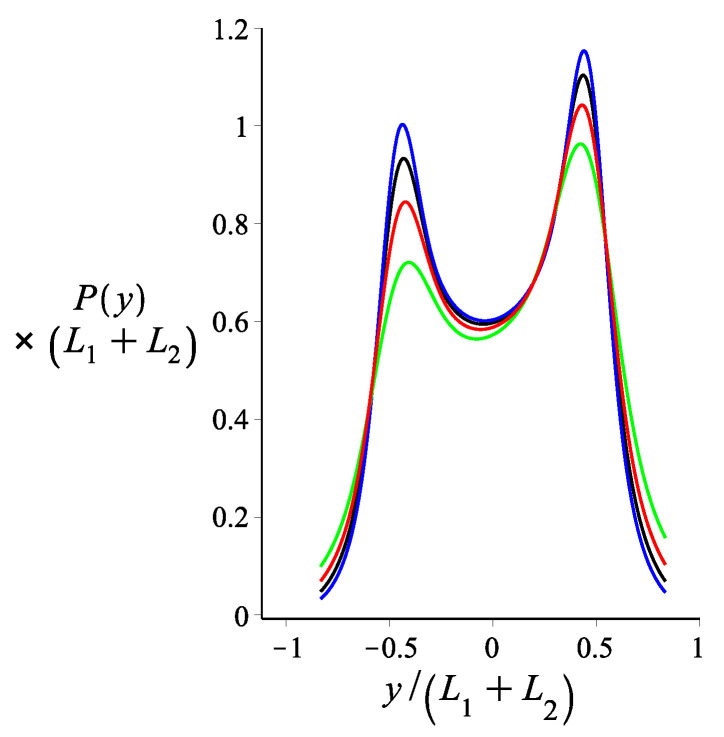
The probability density of the *y* coordinate of the position of the tip of two filaments jointed by a harmonic orientational spring, as shown by Equation (16). The green, red, black and blue colours correspond to $l_{p}=18$ μm, $l_{p}=27$ μm, $l_{p}=36$ μm and $l_{p}=45$ μm, respectively. The fixed parameters for all curves are: $L_{1}=3$ μm; $L_{2}=3$ μm; ω=0; $\theta_{B}=\frac{\pi }{2}$; $\theta_{\mu }=0$ m; $K_{B}=0.5$; $\gamma_{0}=0$; $K_{h}=0.005$; and $l_{p1}=l_{p2}=l_{p}$.

In Figure 6, we show the probability density of the *y* component of the tip position of a magnetic nunchuck when considering different values for the persistence length of the arms. The magnetic interaction breaks the symmetry of the bimodal profile of this probability density due to the specific direction of the magnetic field. The deviation from the symmetrical bimodal profile, measured by the relative difference between the heights of the two peaks, decreases as the persistence length of the arms increases.

**Figure 7:**
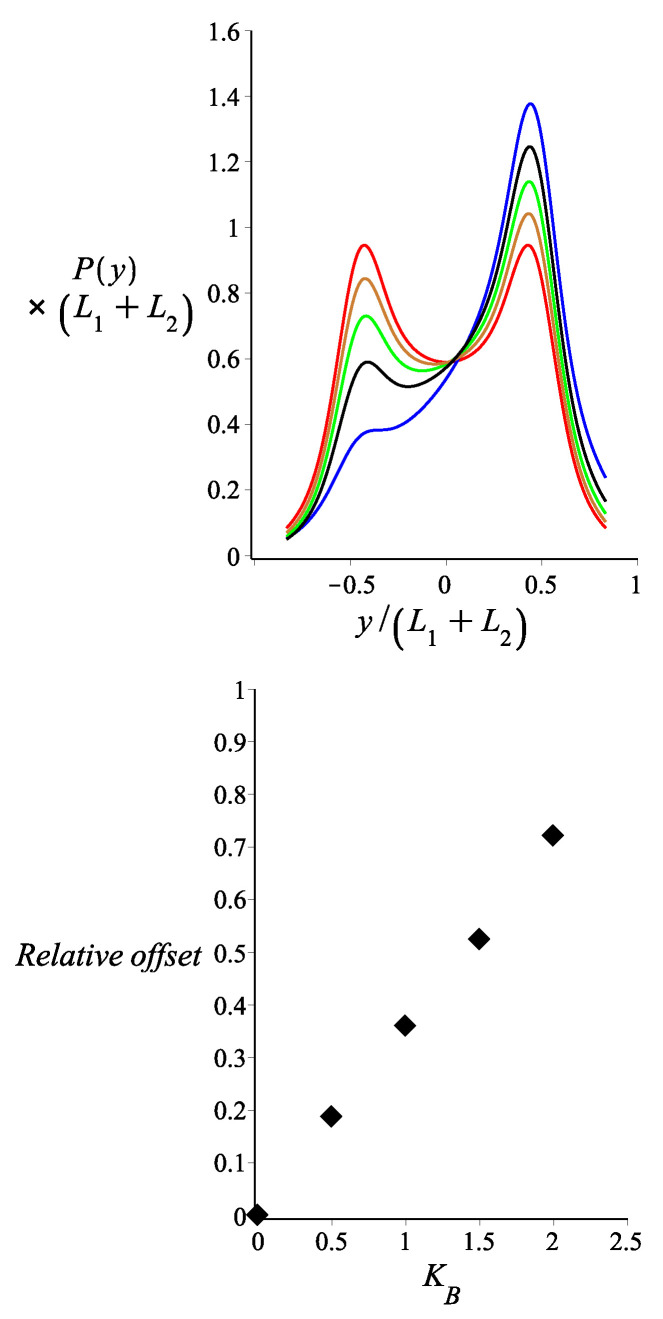
**Upper panel:** The probability density of the *y* coordinate of the position of the tip of two filaments jointed at a hinged point, as shown by Equation (16). The red, gold, green, black and blue colours correspond to $K_{B}=0.005$, $K_{B}=0.5$, $K_{B}=1$, $K_{B}=1.5$ and $K_{B}=2$, respectively. **Lower panel:** The relative offset in the heights of the two peaks in the bimodal profile $\frac{P_{rightmax}-P_{leftmax}}{P_{rightmax}}$ is shown as a function of the magnetic energy $K_{B}$ (measured in units of $k_{B}T$). The fixed parameters for all curves are: $l_{p1}=l_{p2}=l_{p}=27$ μm; $L_{1}=3$ μm; $L_{2}=3$ μm; ω=0; $\theta_{B}=\frac{\pi }{2}$; $\theta_{\mu }=0$; $\gamma_{0}=0$; and $K_{h}=0.005$.

In Figure 7, we show the probability density of the *y* component of the tip position of a magnetic nunchuck for different values of strength of the magnetic energy $K_{B}$. We observe that the profile of the probability density becomes asymmetrical in the presence of the magnetic field in the specific direction. The deviation from the symmetrical bimodal profile increases as the strength of the magnetic energy increases. We measure the deviation from the symmetrical profile by the relative offset in the heights of the two peaks. This is a central result of the present work. We point out that this relative offset plays the role of a very sensitive marker for the magnetic interaction. This sensitivity could be useful in magnetometry. With the appropriate calibration, the magnetic nunchuck could be used as an instrument for the measurement of the magnetic field or for the measurement of the magnetic moment of the bead. As we can see in the lower panel of Figure 7, this method is sensitive to the values of magnetic energy of the order of the thermal energy $k_{B}T$ or even less.

**Figure 8:**
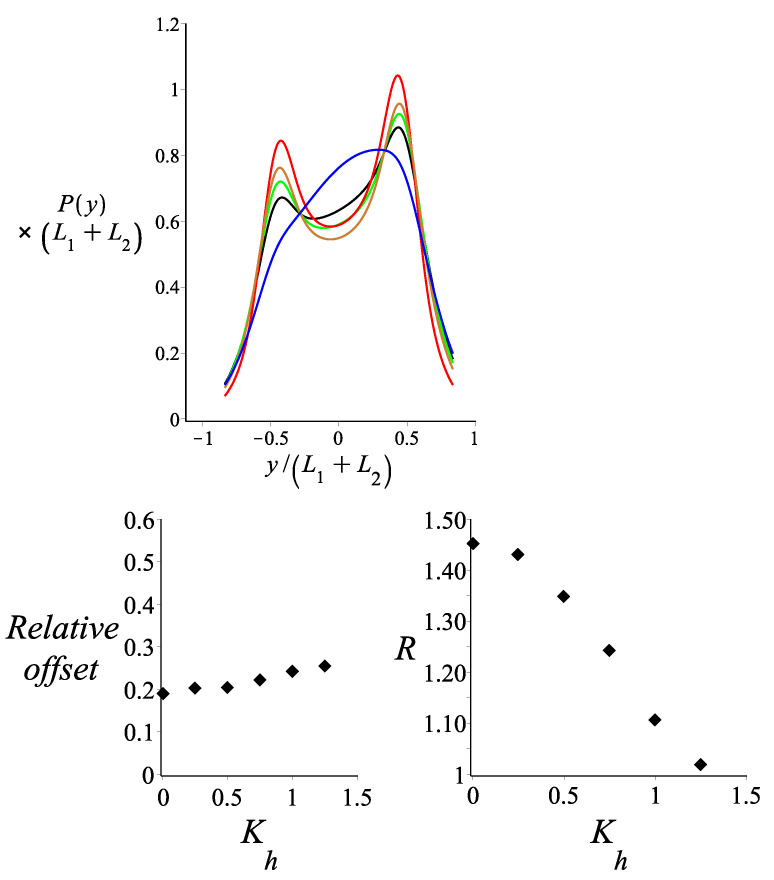
**Upper panel**: The probability density of the *y* coordinate of the position of the tip of two filaments jointed at a hinge point, as shown by Equation (16). The red, gold, green, black and blue colours correspond to $K_{h}=0.005$, $K_{h}=0.5$, $K_{h}=0.75$, $K_{h}=1$ and $K_{h}=2$, respectively. **Lower left panel**: The relative offset of the two peaks of the bimodal profile $\frac{P_{rightmax}-P_{leftmax}}{P_{rightmax}}$ is shown as a function of $K_{h}$. **Lower right panel**: The ratio $\frac{P_{leftmax}}{P_{middlemin}}$ in the bimodal profile is shown as a function of $K_{h}$. The fixed parameters for all curves are: $L_{1}=3$ μm; $L_{2}=3$ μm; ω=0; $\theta_{B}=\frac{\pi }{2}$; $\theta_{\mu }=0$; $K_{B}=0.5$; $\gamma_{0}=0$; and $l_{p1}=l_{p2}=27$ μm.

In Figure 8, the probability density of the *y* component of the tip position of the magnetic nunchuck is shown for different values of the ratio of the stiffness of the hinge point to the thermal energy $K_{h}$. The deviation from the bimodal curve of the probability density increases as the ratio of the stiffness of the hinge point to the thermal energy decreases. The curve tends to become unimodal, with a single peak for the higher values of the stiffness of the hinge point. If the hinge point is viewed as an approximation of a short (relative to the length of the two arms) WLC of contour length $l_{h}$ and persistence length $l_{ph}$, then $K_{h}=l_{ph}/\left(2l_{h}\right)$. The bending stiffness of the hinge $k_{h}$ is related to the bending stiffness of the corresponding WLC segment $\kappa_{h}$ by $k_{h}=\kappa_{h}/l_{h}$. We observe that the asymmetry of the bimodal profile, which is induced by the magnetic interaction, is not very sensitive to the stiffness of the hinge. However, the heights of the peaks relative to the minimum in between is sensitive to the stiffness of the hinge. This sensitivity is also present in the absence of the magnetic interaction, as shown in Figure 4 of Ref. [3].

## 5. Conclusions

In this article, we theoretically analysed the conformations of three grafted semiflexible systems with a magnetic bead at the fluctuating tip. The systems that we considered are confined in two dimensions. All semiflexible parts are treated as weakly bending WLCs. This approximation is justified when the parts are at the stiff limit (e.g., DNA nanorods). For the single filament case, as well as for the case of two filaments jointed by a stiff kink point, we obtained analytic expressions in closed form for the probability density of the *x* and *y* components of the tip position, and also of the tip orientation. For the case of the semiflexible nunchuck, where two weakly bending arms are jointed by a harmonic orientational spring, we obtained analytic expressions up to a single integral. The probability distribution of the transverse (*y*) fluctuations of the tip of the grafted semiflexible nunchuck exhibit a pronounced bimodality, except when the hinge is very stiff. In the absence of a magnetic interaction, the bimodal distribution is symmetrical, but magnetic interaction causes asymmetry. The most remarkable result of our analysis is the sensitivity of this asymmetry, which is quantified by the relative offset in the heights of the two peaks, to the strength of the magnetic interaction. We point out that the relative offset of the two peaks is sensitive to changes in magnetic interaction energy below the thermal energy $k_{B}T$. It is known that, in cantilever magnetometry or other conventional types of magnetometry, thermal fluctuations limit the strength of the signal [8,20]. On the contrary, our system is strongly fluctuating and our method takes advantage of the effect of the magnetic interaction on the conformational fluctuations of the nunchuck. The minimal detectable magnetic moment of an iron-filled carbon nanotube (FeCNT) in a sensitive cantilever experiment was $10^{3}\mu_{B}$ at room temperature and in an external magnetic field of 1T [8]. The ratio of magnetic energy to thermal energy ($k_{B}T$) for that experiment was approximately 2.25. Our proposed method could be more sensitive, at least in theory. The magnetic field-induced relative offset of the two peaks is not very sensitive to the stiffness of the hinge, which in turn depends on the bending stiffness of the linking WLC. On the other hand, the relative height of the bimodal distribution peak to the minimum point in the middle is sensitive to the stiffness of the hinge, irrespective of the magnetic field. This sensitivity could be used, in principle, as an alternative or complementary method for measuring the bending stiffness of the linking WLC segment that acts as a hinge.

## Data Availability

Not applicable.

## Appendix A

### Appendix A.1. The Single Filament

By performing the integrals in Equation (6), we obtain the following result for the probability density of the *x* coordinate position of the tip of the filament attached to the magnetic bead:

(A1) $$P_{x}\left(x\right)=\frac{1}{N_{x}}\exp\left(A_{x}\right)$$

where $N_{x}$ is the normalization factor and

(A2) $$A_{x}=\frac{A_{x}^{\prime }}{A_{x1}^{\prime }}\;,$$

(A3) $$\begin{array}{ccc} A_{x1}^{\prime } & = & 4K_{B}L^{4}\cos\left(3\omega +\theta_{\mu }-\theta_{B}\right)-8K_{B}L^{4}\cos\left(\omega +\theta_{\mu }-\theta_{B}\right)\\ & & +4K_{B}L^{4}\cos\left(\omega -\theta_{\mu }+\theta_{B}\right)+16l_{p}L^{3}\left(\cos\left(2\omega \right)-1\right)\;, \end{array}$$

and

(A4) $$\begin{array}{ccc} A_{x}^{\prime } & = & -2K_{B}^{2}L^{4}\cos\left(2\omega +2\theta_{\mu }-2\theta_{B}\right)\\ & & +K_{B}^{2}L^{4}\cos\left(4\omega +2\theta_{\mu }-2\theta_{B}\right)+8L^{3}K_{B}l_{p}\cos\left(3\omega +\theta_{\mu }-\theta_{B}\right)\\ & & -24L^{2}l_{p}K_{B}x+\cos\left(2\omega +\theta_{\mu }-\theta_{B}\right)+K_{B}^{2}L^{4}\cos\left(2\theta_{\mu }-2\theta_{B}\right))\\ & & +8Ll_{p}K_{B}\left(6x^{2}+L^{2}\right)\cos\left(\omega +\theta_{\mu }-\theta_{B}\right))-48Ll_{p}^{2}x\cos\left(\omega \right)\\ & & +32L^{3}l_{p}K_{B}\cos\left(\omega -\theta_{\mu }+\theta_{B}\right)+24l_{p}^{2}x^{2}+12L_{p}^{2}L^{2}-6L^{4}K_{B}^{2}\\ & & -72L^{2}l_{p}K_{B}x\cos\left(\theta_{\mu }-\theta_{B}\right)+\left(12l_{p}^{2}L^{2}+6L^{4}K_{B}^{2}\right)\cos\left(2\omega \right)\;. \end{array}$$

The result of the integrations in Equation (7) is:

(A5) $$P_{y}\left(y\right)=\frac{1}{N_{y}}\exp\left(A_{y}\right)\;,$$

where $N_{y}$ is the normalization factor and

(A6) $$A_{y}=\frac{A_{y}^{\prime }}{A_{y1}^{\prime }}\;,$$

(A7) $$\begin{array}{ccc} A_{y1}^{\prime } & = & 4K_{B}L^{4}\cos\left(3\omega +\theta_{\mu }-\theta_{B}\right)+8K_{B}L^{4}\cos\left(\omega +\theta_{\mu }-\theta_{B}\right)\\ & & +4K_{B}L^{4}\cos\left(\omega -\theta_{\mu }+\theta_{B}\right)+16l_{p}L^{3}\left(\cos\left(2\omega \right)+1\right)\;, \end{array}$$

and

(A8) $$\begin{array}{ccc} A_{y}^{\prime } & = & -2K_{B}^{2}L^{4}\cos\left(2\omega +2\theta_{\mu }-2\theta_{B}\right)\\ & & +K_{B}^{2}L^{4}\cos\left(4\omega +2\theta_{\mu }-2\theta_{B}\right)+8L^{3}K_{B}l_{p}\cos\left(3\omega +\theta_{\mu }-\theta_{B}\right)\\ & & -24L^{2}l_{p}K_{B}y+\sin\left(2\omega +\theta_{\mu }-\theta_{B}\right)+K_{B}^{2}L^{4}\cos\left(2\theta_{\mu }-2\theta_{B}\right))\\ & & +8Ll_{p}K_{B}\left(6y^{2}+L^{2}\right)\cos\left(\omega +\theta_{\mu }-\theta_{B}\right))+48Ll_{p}^{2}y\sin\left(\omega \right)\\ & & +32L^{3}l_{p}K_{B}\cos\left(\omega -\theta_{\mu }+\theta_{B}\right)-24l_{p}^{2}y^{2}-12L_{p}^{2}L^{2}+6L^{4}K_{B}^{2}\\ & & -72L^{2}l_{p}K_{B}y\sin\left(\theta_{\mu }-\theta_{B}\right)+\left(12l_{p}^{2}L^{2}+6L^{4}K_{B}^{2}\right)\cos\left(2\omega \right)\;. \end{array}$$

### Appendix A.2. The Two Filaments Jointed at a Stiff Kink Point

The probability density of the *y* coordinate position of the end point of the system of two filaments jointed at kink point with the end point attached to a magnetic bead is given by Equation (9). By performing the integrals in Equation (9), we obtain the following closed expression for the probability density:

(A9) $$P_{ky}\left(y_{2}\right)=\frac{1}{N_{ky}}\exp\left(A_{ky}\right)\;,$$

where

(A10) $$\begin{array}{c} A_{ky}=\frac{A_{ky}^{\prime }}{A_{ky1}^{\prime }}\;, \end{array}$$

and

(A11) $$\begin{array}{ccc} A_{ky1}^{\prime } & = & 2K_{B}A_{0}\cos\left(\varphi +\theta_{\mu }-\theta_{B}\right)\\ & & +4\;{l_{p2}}^{2}l_{p1}{L_{1}}^{3}{\left(\cos\left(\omega \right)\right)}^{2}\\ & & +4\;l_{p2}l_{p1}\left({L_{2}}^{3}l_{p1}+3\;{L_{2}}^{2}L_{1}l_{p2}\right){\left(\cos\left(\varphi \right)\right)}^{2}\\ & & +12\;{l_{p2}}^{2}l_{p1}{L_{1}}^{2}\cos\left(\omega \right)L_{2}\cos\left(\varphi \right)\;, \end{array}$$

(A12) φ=ω+γ,

(A13) $$\begin{array}{ccc} A_{0} & = & \left({L_{2}}^{4}{l_{p1}}^{2}+4\;L_{1}l_{p2}l_{p1}{L_{2}}^{3}\right){\left(\cos\left(\varphi \right)\right)}^{2}\\ & & +{L_{1}}^{3}{\left(\cos\left(\omega \right)\right)}^{2}l_{p2}\left(L_{1}l_{p2}+4\;L_{2}l_{p1}\right)\\ & & +6\;{L_{1}}^{2}{L_{2}}^{2}\cos\left(\omega \right)\cos\left(\varphi \right)l_{p1}l_{p2}\;, \end{array}$$

(A14) $$\begin{array}{ccc} A_{ky}^{\prime } & = & {K_{B}}^{2}A_{1}{\left(\cos\left(\varphi +\theta_{\mu }-\theta_{B}\right)\right)}^{2}\\ & & +10\;A_{3}K_{B}l_{P1}l_{p2}\cos\left(\varphi +\theta_{\mu }-\theta_{B}\right)\\ & & +6\;K_{B}{l_{p1}}^{2}l_{p2}{L_{2}}^{2}\mathrm{Zy}\;\cos\left(\varphi \right)\sin\left(\varphi +\theta_{\mu }-\theta_{B}\right)\\ & & +12\;K_{B}l_{p1}L_{2}L_{1}{l_{p2}}^{2}\mathrm{Zy}\;\cos\left(\varphi \right)\sin\left(\varphi +\theta_{\mu }-\theta_{B}\right)\\ & & +6\;K_{B}l_{p1}{L_{1}}^{2}{l_{p2}}^{2}\mathrm{Zy}\;\cos\left(\omega \right)\sin\left(\varphi +\theta_{\mu }-\theta_{B}\right)\\ & & +\left({K_{B}}^{2}{L_{2}}^{4}+3\;{l_{p2}}^{2}{L_{2}}^{2}\right){l_{p1}}^{2}{\left(\cos\left(\varphi \right)\right)}^{2}\\ & & +4\;{K_{B}}^{2}L_{1}{L_{2}}^{3}l_{p1}l_{p2}{\left(\cos\left(\varphi \right)\right)}^{2}\\ & & +6\;{K_{B}}^{2}{L_{1}}^{2}{L_{2}}^{2}\cos\left(\omega \right)\cos\left(\varphi \right)l_{p1}l_{p2}\\ & & +l_{p2}{L_{1}}^{2}\left({L_{1}}^{2}l_{p2}{K_{B}}^{2}+3\;{l_{p1}}^{2}l_{p2}+4\;L_{1}{K_{B}}^{2}L_{2}l_{p1}\right){\left(\cos\left(\omega \right)\right)}^{2}\\ & & -6\;A_{2}{l_{p1}}^{2}{l_{p2}}^{2}\;, \end{array}$$

(A15) $$Zy=L_{2}\sin\left(\varphi \right)-y_{2}+\sin\left(\omega \right)L_{1}\;,$$

(A16) $$\begin{array}{ccc} A_{1} & = & \left({L_{2}}^{4}{l_{p1}}^{2}+4\;L_{1}\;l_{p2}\;l_{p1}\;{L_{2}}^{3}\right){\left(\cos\left(\varphi \right)\right)}^{2}\\ & & +6\;{L_{1}}^{2}{L_{2}}^{2}\cos\left(\omega \right)\cos\left(\varphi \right)l_{p1}\;l_{p2}\\ & & +{L_{1}}^{3}{\left(\cos\left(\omega \right)\right)}^{2}l_{p2}\;\left(L_{1}\;l_{p2}+4\;L_{2}\;l_{p1}\right)\;, \end{array}$$

(A17) $$\begin{array}{ccc} A_{2} & = & L_{2}\left(-y_{2}+\sin\left(\omega \right)L_{1}\right)\sin\left(\varphi \right)+1/2\;{L_{2}}^{2}\\ & & +1/2\;{L_{1}}^{2}+1/2\;{y_{2}}^{2}-L_{1}\sin\left(\omega \right)y_{2}\;, \end{array}$$

and

(A18) $$\begin{array}{ccc} A_{3} & = & \left({L_{2}}^{3}l_{p1}+9/5\;{L_{2}}^{2}L_{1}l_{p2}\right){\left(\cos\left(\varphi \right)\right)}^{2}\\ & & +6/5\;{L_{1}}^{2}l_{p2}\cos\left(\omega \right)L_{2}\cos\left(\varphi \right)\\ & & +\left(L_{1}l_{p2}+3/5\;L_{2}l_{p1}\right){L_{1}}^{2}{\left(\cos\left(\omega \right)\right)}^{2}\\ & & -6/5\;A_{2}\left(L_{2}l_{p1}+L_{1}l_{p2}\right) \end{array}$$
